# An Improved Mobility-Based Control Protocol for Tolerating Clone Failures in Wireless Sensor Networks

**DOI:** 10.3390/s16111955

**Published:** 2016-11-23

**Authors:** Yuping Zhou, Naixue Xiong, Mingxin Tan, Rufeng Huang, Jon Kleonbet

**Affiliations:** 1 Department of Computer Science, Minnan Normal University, Zhangzhou 363000, China; hrf@mnnu.edu.cn; 2 Department of Computer Science, Georgia State University, Atlanta, GA 30302, USA; dnxiong@ieee.org; 3 College of Physical Science and Technology, Central China Normal University, Wuhan 430079, China; tanmingxin@phy.cnu.edu.cn; 4 College of Engineering, Cornell University, Ithaca, NY 14850, USA; Jon.Kleonbet@gmail.com

**Keywords:** emerging sensor networks, sequential test, node clone attacks, mobility-assisted, topology control

## Abstract

Nowadays, with the ubiquitous presence of the Internet of Things industry, the application of emerging sensor networks has become a focus of public attention. Unattended sensor nodes can be comprised and cloned to destroy the network topology. This paper proposes a novel distributed protocol and management technique for the detection of mobile replicas to tolerate node failures. In our scheme, sensors’ location claims are forwarded to obtain samples only when the corresponding witnesses meet. Meanwhile, sequential tests of statistical hypotheses are applied to further detect the cloned node by witnesses. The combination of randomized detection based on encountering and sequential tests drastically reduces the routing overhead and false positive/negative rate for detection. Theoretical analysis and simulation results show the detection efficiency and reasonable overhead of the proposed method.

## 1. Introduction

The technology implementation of multifunctional micro-sensor benefits from the rapid development of Micro-Electro-Mechanism System (MEMS) technology, wireless communications, and System on Chip [1,2,3]. Emerging wireless sensor networks are distributed sensing network architectures constituted by many small, cheap micro-sensors deployed in a monitoring region. These emerging sensor networks have become more and more popular due to their ease of deployment, especially, mobile sensor networks, which include mobile nodes with sensing, communication capacity, and movement ability, are appealing for many applications, for instance, monitoring of animals living in the wild, tracking patients’ heart condition, etc. At the same time, the introduction of the mobile node can also broaden the sampling capacity in the network space [4,5,6,7,8,9], for example, mobile nodes are utilized as information collecting nodes to collect other static nodes’ data in applications [10]. Today mobile wireless sensor networks have been extensively applied in all kinds of applicable fields. For example, mobile information systems with the two functions of mobile communication and mobile computing, are especially suitable for the military operational environment. The solution of the security requirements for mobile wireless sensor networks is highly desired. All kinds of different attacks could be launched by an adversary, which include capture attack, wormhole attack, sinkhole attack, eavesdropping, node clone attacks, etc. Node clone attacks have always been the key issue that affects the security of wireless sensor networks. Because the tiny sensor nodes are arbitrarily deployed and unprotected, generally speaking, these tiny sensor nodes are deployed in locations readily accessible to attackers.

The adversary can capture sensor nodes which lack hardware support for tamper-resistance, and can analyze the captured node for assorted information such as ID, code, key pairs, and then use the credentials of the compromised node to deploy cloned nodes in different strategic locations. The destructiveness would be indefinitely spread throughout the network. Clones with legitimate identities would be able to paralyze the network completely by the way of inside attack, for example, the replicas could not only capture correct data, but also inject false data. They could spy on network traffic, and capture data from the sensor networks. A more serious threat is that the clone could distribute false routing information or silence some nodes to control the network structure [11]. A problem-solving method to avoid cloned nodes is to make nodes tamper-resistant, but the cost implications are prohibitive. Accordingly, detection of cloned nodes is one kind of way to solve the problem effectively.

As far as the location of witness nodes is concerned, there are two kinds of frameworks: centralized detection and decentralized detection. In centralized detection, the data packets including location information are usually forwarded to the base station for detection. To assure the accuracy of detection, the base station must be trusted and powerful. According to the principle of the centralized method, the system has some fatal drawbacks. Firstly, the base station undertaking the arduous task of detecting replicas must be a trusted third party. Once the trusted third party is compromised, a signal peer invalid will appear, and the centralized detection scheme fails. Secondly, nodes surrounding the base station possess undue data communication flaws. Once an adversary damages the communications networks around the witness node, the detection would fail. There is another dimension, which is the power supply of sensor node can easily run out, so the network lifetime is dramatically cut down. Finally the high cost of expensive trusted third parties makes it hard for the centralized detection to be widely used in many wireless sensor networks, so researchers have proposed a new method called distributed detection [12].

In distributed detection, more than one sensor node in different locations acts as witness node, which avoids a possible stumbling block existing in centralized detection. In 2005, Parno et al. [13] presented a distributed scheme called the Randomized Multicast Algorithm for replicas detection. In the presented scheme, the position information of sensor node is broadcast to $\sqrt{n}$ random witness nodes for detection. Parno et al. presented the other method called Line-Selected Multicast, in the presented scheme. The position information of sensor nodes is forwarded to witness nodes which are selected through the analysis of the routing topology. Meanwhile, geometric probability is applied for replica detection. The two protocols share one crucial feature in common, that is the witness nodes selected from networks for detection are random and distributed. In practice, it is hard to achieve the efficiency and security simultaneously in the design of a protocol due to the low success rate of detecting replicas or high communication cost. Therefore, how to select the witness nodes is a dilemma [12]. In particular, it needs a large amount of multi-hop routing overhead to transmit the related information to the witness node for detection in mobile sensor networks. How to reduce routing overhead is another dilemma.

In our study, a novel distributed scheme is presented for detection of node clone attacks, which is called Encounter-based Sequential Hypothesis Testing protocol (ESHT). The basis of the ESHT protocol is the meeting of mobile nodes and the sequential hypothesis testing. In the ESHT scheme, $\sqrt{N}$ random tracked nodes are pre-allocated to each mobile sensor, and every tracked node has $\sqrt{N}$ witness nodes. When the tracked node and its witness node meet, the related location information is transmitted to the witness node, and the witness node judges whether the tracked node is a comprised node or a replica according to whether or not the measured speed *ν* is over the system-configured *ν*_max_. However, this can easily cause many more wrong judgments, if the judgment is made based on only one such observation. To improve the precision, a sequence of speed samples is collected and the Sequential Probability Ratio Test is applied to provide upper bounds for the false positive and false negative rates. Therefore, if two witness nodes with same tracked node encounter, the related detection information is forwarded to one of them and the sequential hypothesis testing method is applied to detect the replica. One major advantage of the ESHT protocol is that the overhead of maintaining a traditional multi-hop routing path is reduced to achieve energy saving effects, and an equally important benefit of the ESHT protocol is that the mobile replicas can be found quickly with a few samples for each tracked node.

The rest of the paper is organized as follows: we describe some of the related studies in Section 2. The Random Waypoint Mobility Model is introduced, which is adopted in our scheme as the mobility mode in Section 3. Section 4 illustrates the system environment, while the encounter-based protocol for detecting mobile clones is presented, which utilizes sequential probability ratio testing. Section 5 describes the theoretical analysis of security and efficiency, and shows our experimental investigation. Finally, the conclusions and guidelines for further research are drawn in Section 6.

## 2. Related Works

In emerging sensor networks, as long as the tiny sensors are arrayed, the position of the sensor remains unchanged. This type of wireless sensor networks is called a static WSN. A commonly used detection principle of node clone attacks in static WSNs is that the same identity with different locations for a sensor node is impossible. This kind of scheme, called claimer-reporter-witness framework is widely adopted to detect static replicated nodes. Two kinds of basic detection methods are often used. One is centralized techniques [14,15,16], the other is decentralized techniques [17,18,19,20,21]. Yu et al. [15] presented an approach utilizing the technology of compressive sensing to distinguish replicas from normal nodes in networks. Zhu et al. [18] described a decentralized method applying Localized Multicast to complete the inspection task. In the study by Zeng et al. [21] for clone detection, two kinds of frameworks called Random Walk and Improved Random Walk based on Table were presented.

Those schemes are not suitable for mobile scenarios due to the continuous movement. When the sensor nodes are mobile, the mobile sensor nodes are not fixed at any specific location. Moreover, it is even harder to forward location claim packets to some witness nodes in a mobile WSN.

In the study by Znaidi et al. [22] a mechanism based on a three-tier hierarchical network structure was used. The principle of the scheme is based on the use of a Bloom filter. The process of detection can be split into three steps: first of all, cryptographic keying materials and some relative parameters are pre-distributed; and secondly, the cluster-head is determined by a relative algorithm; thirdly, the Bloom filter is utilized for the cluster-heads to exchange the ID information, and a node whose ID belongs to more than two clusters is detected as a cloned node. The storage cost of this protocol is significantly reduced. For the additional overhead of Bloom filter and clustering, the communication cost is relatively high. Based on the theory of similarity, the scheme presented in [23] was proposed by utilizing the token-based authentication technology. In the scheme, the broadcast of a timestamp indicates the start of the detection process. Once the detection process starts, a mobile sensor node randomly selects a protected value $S_{i}\in {\left\{0,1\right\}}^{l}$. When a mobile sensor node first encounters another node in the detection period, the two nodes will swap a token with each other, and then save it in their memories. When they meet again in the same detection period, each will request the token exchanged in advance. If the right token is provided, the provider is a genuine node, otherwise the provider is a cloned node. As long as the access between replicas and comprised node is set up by the smart attacker, the token is exposed to replicas, and the scheme fails. In order to prevent conspiracy attack which is launched by communicating with each other, Zhu et al. used a statistics method to detect mobile clones. This principle asserts that a moving node which encounters another node too regularly has probably been captured. Specific counters and lists for recording acquainted nodes are utilized to calculate the total amount of meeting times. The base station is used for centralized analysis.

In the study by Ho et al. [24] the sequential probability ratio test (SPRT) is applied to detect cloned nodes. In the scheme, those mobile sensor nodes whose speed exceeds a predefined speed threshold are detected as replicas. A deadly vulnerability of the scheme introduces an invalid signal peer which is the inherent drawback of centralized techniques. Manickavasagam et al. [25] proposed a distributed scheme based on the optimized SPRT. In the scheme, nodes’ speed is optimized to implement a sequential probability ratio test. At the same time, the message transmission paths are explored to position the intersection. The advantage of the scheme is that higher the node speed, the easier the detection is. Moreover, the presented protocol needs not explicit information packets, but periodic information packets are needed. Its communication cost and storage cost which reach O(n) and O(n) respectively, are relatively reasonable.

Deng et al. [26] presented a method based on a polynomial based on the dispatches of key pairs. In the study, Bloom filters are utilized for verifiable authentication and collection of the total amount of key-pairs which is set up by each mobile node. If the total amount of pair-wise keys set up by the filters is more than the a predefined value, then the nodes are classified as replicas. However, this centralized protocol has a serious flaw. It guarantees nothing about whether the replicas report the right number of keys honestly. Deng et al. [27] presented two decentralized approaches for clone detection in networks. Both of them are based on mobility-assistance. One approach is single storage of time-location claim and exchanges (UTLSE), the principle of the UTLSE protocol is that two nodes exchange related time-location information of the same monitored node until they meet with each other. Then one of the meeting witnesses is selected to detect the cloned node, and only one time-location claim is stored in the UTLSE protocol. Another scheme is called multi-time location storage and diffusion (MTLSD). The principle of MTLSD scheme is similar. The difference is that the second method stores more than one time-location for every monitored node and forwards the information among witnesses. As a result, a higher detection rate can be attained by the MTLSD scheme.

In order to find captured nodes, in the study by Conti et al. [28] two distributed approaches were presented which were called History Information exchange Protocol (HIP) and its optimized version (HOP). Both algorithms are based on the communication with its one-hop neighbor and the mobility. Meanwhile, two kinds of attack modes were defined and their behavior discussed. Their differences are listed as follows: the HIP will keenly observe and analyze node capture attacks only by the information local to the node, but the HOP needs to analyze node cooperation to detect node capture attacks.

A study by Lou et al. [29] depended on the neighborhood community, which can be characterized by the one-hop neighbor node list of the node to be detected. The rationale behind the scheme is that a node cannot appear in different neighborhood communities at any time. The first step of SHP is the fingerprint claim, where the neighbor node table is signed as the passport, and then the passport is broadcasted to one-hop neighbors. If the witnesses receive conflicting passports, there must be replicas present.

Wang et al. [30] presented a study for detecting replicas deployed in wireless sensor networks, whereby some moving nodes act as patrolmen to finish the detection. For static clone nodes, when patrollers migrate to a new region, they spread their patrol information and receive the location messages from surrounding static nodes, and then apply the security thesis that “one benign node only has one location” to detect replicas which have different locations with the same ID. In another interval, the patrollers move to another zone to repeat the same operation. If the clones have been present in a zone, the cloned nodes can be found out once the second conflicting location message is received. In other cases, the cloned node’s answer messages are retrieved by different patrollers, and then they would be detected by the trusted third party or by exchanging information of patrol nodes after around. For the cloned patrol node, the basis of detection is that the speed of moving patrol node should never exceed the predefined maximum speed *V*_max_. When the patroller broadcasts a patrol claim, there will be a static period interval of length T. Once the patroller broadcasts a patrol claim for a new location in time of [*T*,*T* + *interval*], mobile cloned patrollers must exist.

In short, the existing detection schemes based on location confliction are not suitable for mobile wireless sensor networks. The research on detection of node clone attacks working in mobile environments must be different. Due to the mobility of mobile nodes, how to choose the distributed witness becomes a key issue. The cost and difficulty of forwarding related information to the designated witness node are the difficult problem for the solution design.

## 3. Preliminary Information

In mobile wireless networks, node mobility affects the quality of the wireless channel. The dynamic changes of the links between nodes make it harder to design routing protocols [31]. Mobility-assisted protocols based on encounters are put forward to alleviate the situation. The main idea behind the encounter-based protocols is that as a sensor node wants to relay message, it is not required to look for a link to relay the message immediately but to retain the message until the node meets the message recipients or the nodes which enable forwarding the message to the recipients, and then the node forwards the message. This kind of message forwarding way dependent on an encounter-based protocol does not need the overhead of maintaining traditional multi-hop routing paths, and it has been widely used in delay tolerant networks. Research on the statistical characteristics of node meeting, such as the expected meeting time, mean delay time and so on, is of great significance in improving the performance of the protocol due to messaging when only when related nodes meet. Some relevant symbols used in the mobile model definition are given in Table 1. 

There are two definitions given as follows:

**Definition** **1.** *If node i is mobile, whose movement mode is mm, and node j is static, then the hitting probability is the probability that node i hits node j within the time T, which is represented as* $P_{mm}^{H}(t)$*, and* $P_{mm}^{H}(t)=P(\exists \tau \in (0,t]:\left\|X_{i}(\tau )-X_{j}\right\|<R)$.

**Definition** **2.** *If node i and node j are both mobile, their movement mode are mm, then meeting probability is the probability that node i encounters node j within the time T, which is expressed as* $P_{mm}^{M}\left(t\right)$, $P_{mm}^{H}(t)=P(\exists \tau \in (0,t):\left\|X_{i}(\tau )-X_{j}(\tau )\right\|<R)$.

**Lemma** **1.** *In the movement mode based on epoch, if the speed of node is randomly generated in the interval* $\left[v_{\min},v_{\max}\right]$*, where* $v_{\min}>0,v_{\max}<\infty$*, then the mean speed of the node satisfies:*

(1) $$\bar{v}=\frac{v_{\max}-v_{\min}}{\ln\frac{v_{\max}}{v_{\min}}}$$

### 3.1. Random Waypoint Mobility Model

**Definition** **3.** *In the Random Waypoint Mobility (RWP) model, the migration process of each node is as follows: randomly select a waypoint X in the network area; randomly select a value which is in the interval* $[v_{\min},v_{\max}]$ *as movement speed ν; the node moves to waypoint X with speed ν; stops in the waypoint X for a random duration T_stop_ until the epoch is over, where T_stop_ is randomly distributed in the interval* $[0,T_{\max}]$; *repeat the above migration process.*

**Lemma** **2.** If the mobile area of a node is a rectangle, then the Random Waypoint Mobility Model is characterized by the following:

*In one epoch, the mean movement distance for a node is:*

(2) $$\bar{L}\approx 0.5214\sqrt{D}.$$

*The probability that a node locates in location (x,y) is unevenly distributed, and the probability density function is:*

(3) $$f(x,y)\approx \frac{36}{D^{3}}(x^{2}-\frac{D}{4})(y^{2}-\frac{D}{4}).$$

*In one epoch, the mean movement duration is:*

(4) $$\bar{T}=\bar{L}/\bar{v}.$$

*The probability of the movement direction of a node doesn’t obey a uniform distribution. The direction is pointed to the center of the network with high probability. If the center of the network is the origin, then the probability density function of movement direction θ is:*

(5) $$f(\theta )=\frac{1}{4\left|\sin^{3}\theta \right|}(\left|\sin\theta \right|\times g(\theta )+\arcsin(\left|\sin\theta \right|)\times \cos\theta )$$

*where* $g(\theta )=-2\cos^{4}\theta -2\cos^{3}\theta \times \left|\cos\theta \right|+\cos^{3}\times \left|\cos\theta \right|+\cos^{2}\theta +\cos\theta \times \left|\cos\theta \right|+1$.

The location of each node is likely biased towards the center of the network with the movement of the node in the Random Waypoint Mobility model, so this kind of non-uniform distribution of nodes makes it difficult to analyze the Random Waypoint Mobility Model.

#### Hitting Probability

**Lemma** **3.** *In the Random Waypoint Mobility model, the average hitting probability of a node in an epoch is *${\bar{P}}_{rw}^{H}$

(6) $${\bar{P}}_{rw}^{H}=\frac{2R\times \bar{L}}{D}$$

**Proof.**  Node A moves in the Random Waypoint Mobility Model, node B is static in a randomly selected location $X_{B}=(x,y)$ in the network. Further, we suppose that node A locates at the location $X_{s}$ at the beginning of some epoch, and locates at location $X_{f}$ at the end of the epoch, node $X_{B}^{⊥}$ in the line between the node $X_{s}$ and the node $X_{f}$ is the node nearest to the node $X_{B}$. When and only when $\left\|X_{B}-X_{B}^{⊥}\right\|\leq R$, node A hits node B in the current epoch. Suppose all possible collection of epochs can be denoted as $z=\left\{(X_{s},X_{f}):X_{s},X_{f}\in U\right\}$, then all collection of epochs in which node B would be hit can be denoted as $z_{hit|B}=\left\{(X_{s},X_{f}):\|X_{B},X_{B}^{⊥}\|\leq R\right\}$, so the probability that node A hits node B can be calculated by the formula as follow:

(7) $$P_{rw}^{H}(x,y)=\frac{\left\|z_{hit|B}\right\|}{\left\|Z\right\|}=\frac{\left\|z_{hit|B}\right\|}{\underset{U}{\iint }dX_{s}dX_{f}}=\frac{\left\|z_{hit|B}\right\|}{D^{2}}.$$

Analogously, the set of all epochs in which node A is passed through location $X_{B}$ can be denoted as $z_{hit|B}^{*}\{(X_{s},X_{f}):\|X_{B}-X_{B}^{⊥}\|\leq \delta ,\delta \to 0\}$.

The proportion of this kind of epoch to total is ‖zhit|B*‖D2 neglecting the boundary influence. The probability density function of location distribution of node A in random epoch belonging to the set $z_{hit|B}^{*}$ is 1L¯, so the probability density function of node A of location distribution in Random Waypoint Mobility model can be expressed by another formula as follows:

(8) $$f(x,y)=\frac{\left\|z_{hit|B}^{*}\right\|}{D}\times \frac{1}{\bar{L}}$$

In addition, the proportion of the epochs in the set $z_{hit|B}^{*}$ to the epochs in the set $z_{hit|B}$ is 12R, neglecting the influence of boundary, the below equation is derived:

(9) $$\left\|z_{hit|B}\|=2R\times \|z_{hit|B}^{*}\right\|$$

So:

(10) $$P_{rw}^{H}(x,y)=2R\times \bar{L}\times f(x,y)$$

The average hitting probability of node in an epoch is ${\bar{P}}_{rw}^{H}$

(11) $${\bar{P}}_{rw}^{H}=\frac{\iint_{U}P_{rw}^{H}(x,y)dxdy}{\iint_{U}dxdy}=\frac{2R\times \bar{L}}{D}$$

□

### 3.2. Meeting Probability

The probability of the movement direction of a sensor obeys an inhomogeneous distribution in the RWP model. It is more complicated to calculate the standard relative velocity ${\hat{v}}_{rw}$ in the RWP model than in the RDM model. The value of ${\hat{v}}_{rw}$ is given as follows:

**Lemma** **4.** *In Random Waypoint Mobility Model, the standard relative velocity* ${\hat{v}}_{rw}$ *between mobile nodes is about 1.754.*

**Lemma** **5.** *In Random Waypoint Mobility Model, the average of the meeting probability in an epoch is* ${\bar{P}}_{rw}^{M}$:

(12) $${\bar{P}}_{rw}^{M}=\frac{2R\times \bar{L}}{D}(P_{m}\times V_{rw}+2(1-P_{m}))$$

*where* pm=T¯(T¯+T¯stop) *is the probability that a node moves at any time.*

**Proof.**  Node A and node B both move in the RWP model. In the RWP model, the chance that any node is mobile at some moment is $p_{m}$, the probability that any node is static in some moment is $1-p_{m}$, so the probability that node A and node B are both mobile is ${p_{m}}^{2}$; the probability that one node moves but another node remains static is $p_{m}(1-p_{m})$; the probability that both node A and node B are static is ${\left(1-p_{m}\right)}^{2}$. When one node moves but another node remains static, according to Lemma 3, the average of the meeting probability of both nodes in an epoch is

(13) $$p_{rw}^{mp}=\frac{2R\times \bar{v}\times (\bar{T}+{\bar{T}}_{stop})}{D}$$

When both node A and node B are mobile, according to the relativity of motion, one node can be assumed to be static, then another node moves at the speed of ${\hat{v}}_{rw}\times \bar{v}$, the meeting probability of both nodes in an epoch is $p_{rw}^{mm}$:

(14) $$p_{rw}^{mm}=\frac{2R\times {\hat{v}}_{rw}\times \bar{v}\times (\bar{T}+{\bar{T}}_{stop})}{D}$$

When both of node A and node B are static, the meeting probability of both nodes is 0. So the average of meeting probability of both nodes in an epoch is ${\bar{P}}_{rw}^{M}$:

(15) $${\bar{P}}_{rw}^{M}={p_{m}}^{2}\times p_{rw}^{mm}+2p_{m}\times (1-p_{m})\times p_{rw}^{mp}$$

Simplifying:

(16) $${\bar{P}}_{rw}^{M}=\frac{2R\times \bar{L}}{D}(p_{m}\times {\hat{v}}_{rw}+2(1-p_{m}))$$

□

## 4. Protocol Framework

### 4.1. Protocol Requirement

Node replication attacks are very harmful attacks to wireless sense networks. To launch this kind of attack, the attackers need to capture and compromise a legitimate mobile node to get its ID and secret information such as keying materials. Then one or more replica nodes are created by setting the related information of replicas to the same ID and corresponding closet setting of compromised legitimate mobile sensors. The cloned sensors would be deployed in arbitrary locations. During the process of detecting node replication attacks, it is more preferable to utilize distributed monitoring to avoid the inherent drawbacks of centralized monitoring, e.g., single points of failure. At the same time, it is necessary to prevent an attacker from forecasting the witnesses and causing them to fail in advance. Randomized detection, in which the witness nodes are selected randomly, is more secure due to its randomness. The detection protocol should be designed with the characteristics of randomness and distributivity.

The revocation mechanism would be triggered to claim the replica nodes to be illegal if the replica node is detected. In this way, the replica nodes would not be able to communicate with other normal nodes in the mobile wireless sensor networks. Due to their small size, sensor nodes suffer from some inherent drawbacks, e.g., limited power and less storage space which is on the order of a few kilobytes. To improve the detection efficiency of the protocol and reduce the power dissipation and storage usage, the protocol should cut down the overall amount of communication and calculations. At the same time, the performance evaluation indexes used in the protocol are the detection success rate of node clone attacks, communication overhead, and memory overhead.

### 4.2. System and Network Model

A two-dimensional emerging sensor network is constituted by a mass of bulky and cheap sensors which have mobility. Those mobile micro-sensors are arrayed at random in the network, and roam in cyberspace in the light of the Random Waypoint Mobility Model [32].

Every sensor node has the ability to detect its own geographic position. Meanwhile, it can authenticate the situation information of its neighbors. Any secure node localization protocol [33] suitable for the detection of geographic location may be employed. All mobile nodes in the wireless sensor networks use loosely synchronized clocks [34] in a centralized way or in a distributed way.

A general communication model such as bidirectional communication is adopted in the communication link between any two mobile nodes. During the life cycle of mobile sensor networks, the dead nodes whose power has run out and the damaged nodes would be excluded. The base stations in mobile sensor networks can be mobile or static, and must be safe and trusted.

A PKI system [35] is utilized in mobile wireless sensor networks; every node is deployed with a private/public key pair [36]. Every node can obtain other nodes’ public keys from the network authority. The result is that it is nearly impossible for an attacker to forge new identities for sensor nodes in the network. For the sake of detecting node clone attacks, a message freshness mechanism is required to stop replaying attacks in the protocol.

In the system, an adversary has the capacity to catch and conquer a small percentage of legal sensors, and then takes complete control of them to obtain some secret information including private keys, credentials and cryptographic information. The adversary can operate with legal status in the network after obtaining the private information. They can mount all kinds of attacks, e.g., they can eavesdrop on packets, inject false data, and break supported protocols including sensor node localization protocols, clock synchronization protocols, and message freshness mechanisms. In addition, it is easy for replica nodes to launch denial-of-service attacks by way of deleting data packets from a benign node. Supposing the ability of the attacker to subvert legitimate sensor nodes is limited, then only a limited number of legitimate sensor nodes would be conquered. If the vast majority of legal sensor nodes are subverted, any scheme for detecting node replication attacks may then become defunct in the network. We are also working on the assumption that at least a one-hop neighbor of the clone is benign. The adversary captures and compromises benign node behind the closed doors in complete secrecy to avoid touching off automated detection for node replication attacks. At the same time, attackers can remember the subverted nodes and do not repeat to compromise the same nodes.

### 4.3. Sequential Probability Ratio Test

In a static network environment, it would be illegal for a static node to appear in different locations, so a static sensor node is reasoned to be a replica node according to its appearance in more than one place. When the sensor node is mobile, a legal node would be falsely regulated as a cloned node or a captured node, so the judgment on whether a sensor is a replica node cannot be dependent on the technique. We must try to search for other techniques for cloned node detection in mobile environments. According to the mobility property of the sensor nodes, a normal node would not be able to move beyond some maximum speed $v_{\max}$ which can be scientifically configured by the system. On the contrary, a replica nodes’ measured speed would be faster than the ordinary speed, it would even appear to be over the speed threshold, provided the adopted velocity measuring system possesses a low error rate, since there are two or more than two mobile nodes with the same identity in different places at once. Without loss of generality, provided that the speed of a mobile node exceeds the predefined value, there is therefore a high chance that the node is detected as a cloned node. That is, a high speed exceeding $v_{\max}$ means that two or more than two mobile nodes which are subverted or cloned are found out to coexist in the sensor network.

Due to the clue of maximum speed, the method of Sequential Tests of Statistical Hypotheses [37] has been proposed to solve the detection of node replication attacks. Wald first put forward the theory of the Sequential Probability Ratio Test (SPRT). In fact SPRT is a particular statistical model. In 1933, Wald further proposed a sequential analysis problem using as inspiration the results reported by Neyman and Pearson [38].

A new detection method for node replication attacks which leverages a Sequential Probability Ratio Test is proposed. According to the Sequential Tests of Statistical Hypotheses, the test is assumed to be a direct line random walk between the prescribed minimum pointer and the prescribed maximum pointer. At the start, the prescribed minimum is linked to the null hypothesis. On the other hand, the prescribed maximum is linked to the alternate hypothesis. When the test begins, the random walk moves from an arbitrary point on the direct line toward the two endpoints including the lower limit and upper limit. Its movement is in the light of the measured speed of a moving sensor node. The lower limit should be constructed by linking with a speed that is below the predefined maximum value $v_{\max}$, and the upper limit should be considered to exceed $v_{\max}$, respectively. Each time the random walk hits or crosses the prescribed minimum, then the null hypothesis is accepted, that is the mobile sensor node is detected as a normal node. For another aspect, when the random walk hits or crosses the prescribed maximum, then the alternate hypothesis are accepted, that is the mobile sensor node is detected as a replica node or captured node.

The basic principle of Sequential Tests of Statistical Hypotheses which is applied for the mobile replica detection can be described as follows: as a moving node migrates to a new position, it is necessary to judge whether the meeting one-hop neighbors are its witness node. If the meeting neighbors are just the witness node, each of the neighboring witnesses asks for a signed time-location claim and decides whether to store the received claim probabilistically. The witness node computes the speed by the application of two consecutive time-location claims of its tracked mobile node. Here, every speed is considered as an analysis sample of Sequential Tests of Statistical Hypotheses. If the sample exceeds the system-configured speed $v_{\max}$, the random walk would be expedited in the direction of the prescribed minimum. Once the roam hits or crosses the prescribed maximum, and then node replication attacks are detected. On the other hand, if the observed speed does not exceed the maximum speed $v_{\max}$, the random walk would be promoted along the lower limit. Once the random walk hits or crosses the lower limit, the null hypotheses would be accepted, that is the mobile node is a normal node.

### 4.4. Methods

Different from those methods for detecting cloned nodes in networks, in which the relevant detection information is transferred to the designed witness node for detection, here a novel proposed protocol which is called encounter-based sequential hypothesis testing protocol (ESHT) does not require related routing algorithms and routing messages for the path-finding of witness nodes. The mobility feature of sensor nodes is utilized to realize the protocol. When the tracer, that is witness node, obtains time-location information of one-hop neighbor which is just in its tracking set, the Sequential Tests of Statistical Hypotheses are applied for detecting cloned nodes in the mobile network. As soon as two witness nodes which have the same tracked node encounter each other, the detection information is transferred to one of the meeting witness nodes for detection. Table 2 shows correlative notations used in the ESHT. 

There are three stages in the ESHT protocol as follows:

#### 4.4.1. Claim Generation and Verification

In the deployment process, every mobile node is initially associated with a set of traced nodes. Each node is the witness node of all nodes in its tracking set. That is, the node is the tracer of all nodes in its own tracking set. To avoiding overloading the tracer and taking advantage of the meeting chances of nodes, it is reasonable to make tracking set scale equal to $\sqrt{N}$, because the probability that the encountering nodes have at least one same tracked node according to the Birthday Paradox is 50%. When a moving sensor node L migrates to a new position and encounters a new neighboring mobile node, and if the mobile node is in the tracking set of the neighboring node, the neighboring node requires for verifiable time-location claim by sending a request packet including current time $T_{n}$ to the requested node L. Node L would discard the request, once the following condition holds

(17) $$\left|T_{L}-T_{n}\right|>\xi +\tau$$

where $T_{L}$ is the current time that mobile sensor node L receives the request; $T_{n}$ is the time that neighboring node starts the request; ξ is the transmission delay of time-location claim; τ denotes a maximum error during the process of temporal synchronization. Because the transmission distance is over transmission radius of a node, the time-location claim is not from the neighboring node. Otherwise, node L generates its time-location claim which can be expressed as $\left\{ID_{L},t_{L},l_{L},{\mathrm{SIG}}_{{\mathrm{SK}}_{L}}(H(ID_{L}||t_{L}||l_{L}))\right\}$, where $ID_{L}$ is the identity of mobile sensor L, and $l_{L}$ is node *L* position, $t_{L}$ is the time of generating this time-location claim; ‖ indicates the concatenation operation, and ${\mathrm{SIG}}_{{\mathrm{SK}}_{L}}(H(ID_{L}||t_{L}||l_{L}))$ denotes encrypting the hash value of the data that performs a cascade of the ID, the time-position claim generation time and the position of node L by utilizing the private key of node L for the sake of implementing authentication of node L. The neighboring node which acts as the tracer of node L would validate the data integrity of the received data packet by utilizing the public key of node L and validate the plausibility of the distance between the tracer and tracked node L. If the verification fails, the time-location claim would be ignored, whereas the claim would be preserved with probability p.

#### 4.4.2. Encountering and Detection

When one mobile node a encounters some node L which belongs to the tracking set of node a once more, the claim generation and verification procedure are repeated. The mobile node would receive more than one time-location claim which would be denoted by $C_{L}^{1},C_{L}^{2}\ldots$, and then extract interrelated information such as the time information $t_{L}^{i}$ and the location information $l_{L}^{i}$ from claim $C_{L}^{i}$. On the basis of Euclidean distance, the Euclidean distance between the point $l_{L}^{i}$ and the point $l_{L}^{i+1}$ could be computed:

(18) $$d_{L}^{i}=sqrt(\sum_{j}{\left(x_{L_{i+1}}^{j}-x_{L_{i}}^{j}\right)}^{\wedge }2)$$

where $x_{L_{i+1}}^{j}$ denotes the *j*th dimensional coordinate of the point $l_{L}^{i+1}$, $x_{L_{i}}^{j}$ denotes the *j*th dimensional coordinate of the point $l_{L}^{i}$. Let the measured speed $v_{L}^{i}$ at time $T_{i+1}$ be a trial sample in a Bernoulli trial:

(19) $$v_{L}^{i}=\frac{d_{L}^{i}}{\left|t_{L}^{i+1}-t_{L}^{i}\right|}$$

The Bernoulli random variable is denoted by $S_{L}^{i}$:

(20) $$S_{L}^{i}=\{\begin{array}{ll} 0, & if\;v_{L}^{i}\leq v_{\max}\\ 1, & if\;v_{L}^{i}>v_{\max} \end{array}$$

When the measured speed is over $v_{\max}$, it indicates the mobile node is a cloned node and $S_{L}^{i}$ is set to 1. Instead, when the measured speed is not over $v_{\max}$, it indicates the mobile node is a normal node and $S_{L}^{i}$ is set to 0. The probability of success is expressed as α:

(21) $$\alpha =\mathrm{Pr}(S_{L}^{i}=1)=1-\mathrm{Pr}(S_{L}^{i}=0)$$

There a pre-defined threshold $\alpha^{\prime }$ is used to judge whether node L is a replica. If the probability of success α is more than or equal to the predefined threshold $\alpha^{\prime }$, then it can be inferred that the mobile node L is a cloned node. Conversely, when the success rate α is below the predefined threshold $\alpha^{\prime }$, the moving node is considered a normal node. The problem of detecting node replication attacks can be reduced to a sequential probability ratio test. In this test, a good sampling strategy is adopted to tolerate the maximum chance errors. To do it, the sequential probability ratio test can be further reduced to a test which contains null hypotheses and alternate hypotheses of $\alpha \leq \alpha_{0}$ and $\alpha \geq \alpha_{1}$ respectively, where $\alpha_{0}\leq \alpha_{1}$. When the null hypotheses is accepted and $\alpha \geq \alpha_{1}$, this will cause false negative error. On the other hand, When the alternate hypotheses is accepted and $\alpha \leq \alpha_{0}$, this will result in false positive error. In order to try to void those two error types, the maximum false negative rate γ and the maximum false positive rate η are configured as the threshold to guarantee that the false negative rate is not more than γ and the false positive rate does not exceed η.

The sequential probability ratio test defines two kinds of hypotheses, one is the null hypothesis $H_{0}$, another is the alternate hypothesis $H_{1}$. The null hypothesis means the node is normal. On the contrary, the alternate hypothesis means the node is a clone. In the sampling plan, the measured speed is the sample. It is an important problem that how to judge whether the mobile node has been cloned according to the observed n samples. To comprehend the principle of the sampling scheme, the logarithmic probability ratio on n sample is defined as $L_{n}$:

(22) $$L_{n}=\ln\frac{\mathrm{Pr}(S_{L}^{l},\cdots ,S_{L}^{n}|H_{1})}{\mathrm{Pr}(S_{L}^{l},\cdots ,S_{L}^{n}|H_{0})}$$

In the Bernoulli experiment, each sample is measured independently, so $S_{L}^{i}$ is i.i.d. random variable sequences. Therefore Equation (22) can be reformulated to obtain:

(23) $$\begin{array}{ll} L_{n} & =\ln\frac{\prod_{i=1}^{n}\mathrm{Pr}(S_{L}^{i}|H_{1})}{\prod_{i=1}^{n}\mathrm{Pr}(S_{L}^{i}|H_{0})}\\ & =\sum_{i=1}^{n}\ln\frac{\mathrm{Pr}(S_{L}^{i}|H_{1})}{\mathrm{Pr}(S_{L}^{i}|H_{0})} \end{array}$$

Let $\omega_{a}^{L}$ represent the cumulative total of the number of times a specific event that $S_{L}^{i}=1$ occurs in the $n_{a}^{L}$ samples of the tracked node L whose witness node is node a, then Equation (23) can be reformulated to obtain:

(24) $$L_{n}=\omega_{a}^{L}\times \ln\frac{\alpha_{1}}{\alpha_{0}}+(n_{a}^{L}-\omega_{a}^{L})\times \ln\frac{1-\alpha_{1}}{1-\alpha_{0}}$$

where $\alpha_{0}=\mathrm{Pr}(S_{i}|H_{0})$ and $\alpha_{1}=\mathrm{Pr}(S_{i}|H_{1})$, the fundamental principle of the parameter setting of $\alpha_{0}$ and $\alpha_{1}$ is described below: $\alpha_{0}$ should be set according to the possibility of the event that a normal node is misjudged as cloned node due to time synchronization and localization errors. Another dimension to consider is the fact that $\alpha_{1}$ should be configured according to the possibility of the event that the measured speed of a cloned node is over the predefined maximum value $v_{\max}$. On the basis of above analysis, the former should be less than the later.

Sequential probability ratio test takes advantage of the log-probability ratio $L_{n}$ to determine whether to accept the hypothesis $H_{0}$ or the hypothesis $H_{1}$ or not. The decision process is given as follows:

$L_{n}\leq \ln\frac{\gamma }{1-\eta }$; The hypothesis $H_{0}$ is correct and the detection is over;
$L_{n}\geq \ln\frac{1-\gamma }{\eta }$; The hypothesis $H_{1}$ is correct and the detection is over;
$\ln\frac{\gamma }{1-\eta }<Ln<\ln\frac{1-\gamma }{\eta }$; Continue the detection process with sample,

where γ is the maximum false negative rate, and η is the maximum false positive the next rate. Then we substitute Equation (24) into the decision process to reformulate the process:

$\omega_{a}^{L}\leq \Psi_{0}(n_{a}^{L})$; The hypothesis $H_{0}$ is correct and the detection is over;
$\omega_{a}^{L}\geq \Psi 1(n_{a}^{L})$; The hypothesis $H_{1}$ is correct and the detection is over;
$\Psi_{0}(n_{a}^{L})<\omega_{a}^{L}<\Psi 1(n_{a}^{L})$; Continue the detection process with the next sample,

where $\Psi_{0}(n_{a}^{L})=\frac{\ln\frac{\gamma }{1-\eta }+n_{a}^{L}\times \ln\frac{1-a_{0}}{1-a_{1}}}{\ln\frac{a_{1}}{a_{0}}-\ln\frac{1-a_{1}}{1-a_{0}}}$, $\Psi_{1}(n_{a}^{L})=\frac{\ln\frac{1-\gamma }{\eta }+n_{a}^{L}\times \ln\frac{1-a_{0}}{1-a_{1}}}{\ln\frac{a_{1}}{a0}-\ln\frac{1-a_{1}}{1-a_{0}}}$ At the same time, the detection information $I_{a}^{L}=(n_{a}^{L},\omega_{a}^{L})$, which belongs to the tracked node L whose tracer is node a, is stored in a queue associated with the node L. Once a mobile node is detected as a replicated node, then the witness node makes use of broadcast security protocol [39] to notify all nodes in the wireless sensor networks to ignore the malicious node. The protocol is over, otherwise, the protocol proceeds to the third phase, the forwarding and detection stage.

#### 4.4.3. Forwarding and Detection

When one mobile node a encounters a mobile node b, and the two nodes have the same tracked nodes, in other words, $D_{a}\cap D_{b}\neq \emptyset$ ($D_{x}$ is the tracking set of node x). If $ID_{a}>ID_{b}$, for $\forall x\in D_{a}\cap D_{b}$ node a submits a testing request to node b. A testing request $R_{a}^{x}$ involves $\left\{n_{a}^{x},\omega_{a}^{x},t_{a},{\mathrm{SIG}}_{{\mathrm{SK}}_{a}}(H(ID_{a}||n_{a}^{x}||\omega_{a}^{x}||t_{a}))\right\}$, where $n_{a}^{x}$ represents the total number of samples of tracked node x whose witness node is node a; $\omega_{a}^{x}$ represents the cumulative total of the amount of times a specific event that $S_{x}^{i}=1$ occurs in the $n_{a}^{x}$ samples of the tracked node x whose witness node is node a; $t_{a}$ represents the time in which the detection request is submitted to node b. ${\mathrm{SIG}}_{{\mathrm{SK}}_{a}}(H(ID_{a}||n_{a}^{x}||\omega_{a}^{x}||t_{a})$ denotes encrypting the hash value of the data which performs a concatenation of the ID of node a, the total number of samples of node x, the total number of times an event has occurred that $S_{x}^{i}=1$, and the time of starting to send the detection request by utilizing the private key of node a for the sake of implementing integrity verification of the message. In Bernoulli experiment, every measured speed sample is independent, once node b receives the detection request from node a, the total number of speed samples of the tracked node x is $n^{x}$:

(25) $$n^{x}=n_{a}^{x}+n_{b}^{x}$$

$n^{x}$ is the sum of the number of speed samples of the tracked node x whose witness nodes are node a and node b, respectively. Let $\omega^{x}$ represent the cumulative total of the number of times a specific event that $S_{x}^{i}=1$ occurs in the $n^{x}$ samples of the tracked node x:

(26) $$\omega^{x}=\omega_{a}^{x}+\omega_{b}^{x}$$

Substituting Equations (25) and (26) into Equation (24) we obtain:

(27) $$Ln=\omega^{x}\times \ln\frac{\alpha_{1}}{\alpha_{0}}+(n^{x}-\omega^{x})\times \ln\frac{1-\alpha_{1}}{1-\alpha_{0}}$$

Then we apply Equations (25)–(27) in the second stage of the decision process to reformulate the process:

$\omega_{a}^{x}+\omega_{b}^{x}\leq \Psi_{0}(n_{a}^{x}+n_{b}^{x})$; The hypothesis $H_{0}$ is correct and the detection is over;
$\omega_{a}^{x}+\omega_{b}^{x}\geq \Psi 1(n_{a}^{x}+n_{b}^{x})$; The hypothesis $H_{1}$ is correct and the detection is over;
$\Psi_{0}\times (n_{a}^{x}+n_{b}^{x})<\omega_{a}^{x}+\omega_{b}^{x}<\Psi 1\times (n_{a}^{x}+n_{b}^{x})$; Continue the detection process with the next sample,

where $\Psi_{0}(n^{x})=\frac{\ln\frac{\gamma }{1-\eta }+(n_{a}^{x}+n_{b}^{x})\times \ln\frac{1-\alpha 0}{1-\alpha 1}}{\ln\frac{\alpha 1}{\alpha 0}-\ln\frac{1-\alpha 1}{1-\alpha 0}}$, $\Psi_{1}(n_{a}^{L})=\frac{\ln\frac{1-\gamma }{\eta }+(n_{a}^{x}+n_{b}^{x})\times \ln\frac{1-\alpha 0}{1-\alpha 1}}{\ln\frac{\alpha 1}{\alpha 0}-\ln\frac{1-\alpha 1}{1-\alpha 0}}$.

To accelerate the speed of detection, an intuition is to maintain one queue for each node in the tracking set, which can hold more than two pieces of corresponding detection information. The more samples are measured, the more accurate the detection, but the longer the queue, the higher the space costs, so making the size of each queue equal to 3 would be meeting demand. As soon as two nodes with the same tracked node meet, for example, node a meets node b (if $ID_{a}>ID_{b}$), for $\forall x\in D_{a}\cap D_{b}$, node a submits a testing request to node b. There is a queue Q for node x including three relational detection information in node b, if $I_{a}^{x}$ already exits, the latest $I_{a}^{x}$ updates the existing $I_{a}^{x}$. If $I_{a}^{x}$ doesn’t exit and there is some space available in the queue, then the received $I_{a}^{x}$ should be written into the queue. If $I_{a}^{x}$ doesn’t exit and there is no space available in the queue, then the received $I_{a}^{x}$ should overwrite the detection information existing longest in the queue for node x. In the process of detection, assume there are three detection information $I_{a}^{x}$, $I_{b}^{x}$ and $I_{c}^{x}$ in the queue, the total number of speed samples for the tracked node x, $n^{x}=n_{a}^{x}+n_{b}^{x}+n_{c}^{x}$, $\omega^{x}=\omega_{\alpha }^{x}+\omega_{b}^{x}+\omega_{c}^{x}$, substitute $n^{x}$ and $\omega^{x}$ into the decision process.

Once a mobile node is detected as a replicated node, then the witness node makes use of broadcast security protocol to notify any other nodes in the network to dismiss the malicious node.

## 5. Performance Analysis

The detection probability is the probability that the replica nodes are accurately detected. The effect of different amount of epochs on the success rate has been an important performance index. Here an epoch is a random time interval, in this time interval a node keeps moving in the identical direction and at a constant velocity. The movement pattern adopted in the proposed protocol is Random Waypoint Mobility Model. Some random characteristics of RWP are adopted in the security analysis. In the following, the lower bound of detection probability would be analyzed.

**Theorem** **1.** *Assume node* e *is a compromised node, and* $e_{1}$ *is a cloned node of* e*,* e *runs into one of its witness node with the time-location claim* $\left\{ID_{e},t_{e},l_{e},{\mathrm{SIG}}_{{\mathrm{SK}}_{e}}(H(ID_{e}||t_{e}||l_{e}))\right\}$*,* $e_{1}$ *encounters the same witness with the time-location claim* $\left\{ID_{e1},t_{e1},l_{e1},{\mathrm{SIG}}_{{\mathrm{SK}}_{e1}}(H(ID_{e1}||t_{e1}||l_{e1}))\right\}$*, then the chance of the witness node detecting the node replication attacks by utilizing the two time-location claims is*:

(28) $$P_{d}(t)=1-\frac{\pi }{D}\times {\left(v_{\max}\times t\right)}^{2}$$

in which $t=|t_{e}-t_{e1}|$.

**Proof.**  Assume f(x,y) is the probability density function that a node appears in a position (x,y) in the networks, and nodes in the network are uniformly distributed, therefore f(x,y)=1/D. Node e is in the position (xe,ye), the conditional probability that node e and node $e_{1}$ are contradictory in their locations is:

(29) $$P_{d}(t|l_{e}=(x_{e},y_{e}))=\iint_{U}f(x,y)dxdy$$

where $U=\{(x,y)\in S|\sqrt{{\left(x-x_{e}\right)}^{2}+{\left(y-y_{e}\right)}^{2}}>v_{\max}\times t\}$, and:

(30) $$\begin{array}{ll} P_{d}(t) & =P_{d}(t|l_{e})P(l_{e})\\ & =P(\left|l_{e}-l_{e_{1}}\right|>v_{\max}\times t)\\ & =\iint_{U}f(x_{e},y_{e})\times P\times d(t|l_{e}=(x_{e},y_{e}))dx_{e}dy_{e} \end{array}$$

Then:

(31) $$P_{d}(t)=1-\frac{\pi }{D}\times {\left(v_{\max}\times t\right)}^{2}$$

□

Assume there are only two nodes with the same identity, they are the compromised node e and its cloned node $e_{1}$. Only one witness node is pre-distributed to detect the node replicas attacks. Let $N_{d}$ denote the amount of epochs until the witness node detects the replica sensor. $N_{m}$ indicates the number of epochs that is the only time the witness node encounters malicious node before the witness node detects the replica node, then:

(32) $$P(N_{m}=i)=\left(\begin{array}{c} 2\\ 1 \end{array}\right){\left(1-p\right)}^{i-1}p{\left(1-p\right)}^{k-i}=\left(\begin{array}{c} 2\\ 1 \end{array}\right){\left(1-p\right)}^{k-1}p$$

where p is the probability that any two nodes encounter in one epoch.

Let $P(N_{d}=k)$ indicate the chance that the node replication attacks are found out until the *k*th epoch, only if the witness node receives two time-location claims coming from the different nodes with the same identity, the malicious nodes can be detected with a specified probability, and half time-location claims are useful. The probability:

(33) $$P(N_{d}=k)=\frac{1}{2}\sum_{i=1}^{k}\sum_{j=i}^{k}P(N_{m}=i)P(N_{m}=j)P_{d}(i,j)$$

where $P_{d}(i,j)$ denotes the probability that the witness node detects node replication attacks when the witness node encounters node e at *i*th epoch and encounters node $e_{1}$ at *j*th epoch. According to Theorem 1, $P_{d}(t)$ represents the probability that the witnesses detect node replication attacks by utilizing the two time-location claims received from e and $e_{1}$ respectively. When the two time-location claims are retrieved in different epoch, $P_{d}(t)$ is 0. In the meantime, the time $t_{e}$ and $t_{e_{1}}$ which are included in the two time-location claims are assumed to obey the uniform distribution in the same epoch, so the average difference of the two times is:

(34) $$E(\left|t_{e}-t_{e_{1}}\right|)=\frac{\bar{T}+{\bar{T}}_{stop}}{3}$$

Therefore:

(35) $$P_{d}(i,j)=\{\begin{array}{cc} P_{d}\times ((\bar{T}+{\bar{T}}_{stop})/3) & ,i=j\\ 0 & ,i\neq j \end{array}$$

Then we substitute Equations (28) and (35) into Equation (33) to obtain:

(36) $$P(N_{d}=k)=2^{\left(2k-1\right)}\times {\left(1-p\right)}^{2(k^{2}-k)}\times p^{2k}\times (1-\frac{\pi }{D}\times {v_{\max}}^{2}\times \frac{{\left(\bar{T}+{\bar{T}}_{stop}\right)}^{2}}{9})$$

After n epochs, the witness node detects the two malicious nodes with the following probability:

(37) $$P_{2}(n)=\sum_{k=1}^{n}\left(\prod_{\omega =0}^{\kappa -1}\left(1-P(N_{d}=w)\right)\times P(N_{d}=k\right))\;P_{2}(n)=\sum_{k=1}^{n}P(N_{d}=k)$$

The real probability that one witness node detects replicas is larger than $P_{2}(n)$, since the witness nodes have some probability of sampling more than one measured speed before the *k*th epoch but until the *k*th epoch, the witness node detects the node replication attacks.

In the proposed scheme, each node has $\sqrt{N}$ witness nodes, the detection probability after *n* epochs in step 2 is $P_{d2}(n)$:

(38) $$P_{d2}(n)\geq 1-{\left(1-P_{2}(n)\right)}^{\sqrt{N}}$$

When the node replication attacks are not detected at the second stage of the detection protocol, the protocol proceeds to the third phase. We assume there are only two witness nodes which would detect the compromised node e and its cloned node $e_{1}$. Let ${N_{m}}^{\prime }$ denote the number of epochs before the malicious node is detected in the second phase of the protocol. Let $P({N_{m}}^{\prime }=k)$ represent the probability that the node replication attacks are not detected until the *k*th epoch in the second stage of the protocol, under the premise that there is one speed sample gained from the malicious nodes:

(39) $$P({N_{m}}^{\prime }=k)=\frac{1}{2}\sum_{i=1}^{k}\sum_{j=i}^{k}P(N_{m}=i)\times P(N_{m}=j)\times (1-P_{d}(t))$$

$P_{3}(n)$ represents the maximum probability that the witness node detects the two malicious nodes in the third phase of the protocol after n epochs:

(40) $$P_{3}(n)=\sum_{k=1}^{n}p\times {\left(1-p\right)}^{k-1}\times P({N_{m}}^{\prime }=k)$$

where p is the probability that any two mobile nodes encounter in an epoch. According to the principle of Sequential probability ratio test, we assume that $p_{fp}$ is the false positive rate and $p_{fn}$ is the false negative rate, in the light of Wald’s theory [40], the predefined maximum boundary of $p_{fp}$ is computed by $p_{fp}\leq \frac{\eta }{1-\gamma }$. Similarly the truth, the predefined maximum boundary of $p_{fn}$ is computed by $p_{fn}\leq \frac{\gamma }{1-\eta }$. The sum of the false positive rate $p_{fp}$ and the false negative rate $p_{fn}$ meets the following inequality:

(41) $$p_{fp}+p_{fn}\leq \gamma +\eta$$

Since $p_{fn}$ is the false negative, therefore the detection probability for node replication attacks is $1-p_{fn}$. The lower bound of $1-p_{fn}$ is given as follows:

(42) $$1-p_{fn}\geq \frac{1-\gamma -\eta }{1-\eta }$$

Because each witness node has some probability to sample more than one measured speed before the *k*th epoch, and the witness node detects the node replication attacks until the *k*th epoch, so the real probability that one witness node detects replicas is larger than $P_{3}(n)$. Let ${P_{d3}}^{\prime }(n)$ denote the probability that the node replication attacks are detected in the third stage of the protocol when there are only two witness nodes. Then we can substitute the lower bound of replica detection probability into Equation (40) to obtain:

(43) $$\begin{array}{l} {P_{d3}}^{\prime }(n)\geq P_{3}(n)\times (\frac{1-\gamma -\eta }{1-\eta })\\ \;\geq \sum_{k=1}^{n}p\times {\left(1-p\right)}^{k-1}\times P({N_{m}}^{\prime }=k)\times (\frac{1-\gamma -\eta }{1-\eta }) \end{array}$$

Considering the balance between storage cost and detection efficiency, every node has $\sqrt{N}$ witness nodes, and thus the detection probability after n epochs in step 3 is:

(44) $$P_{d3}(n)\geq 1-{\left(1-{P_{d3}}^{\prime }(n)\right)}^{\left(\begin{array}{c} \sqrt{N}\\ 2 \end{array}\right)}$$

The metrics used to evaluate efficiency of the proposed protocol are:

- Communication overhead: the average amount of packets which are transmitted and received by each node when the protocol for detecting node clone attacks works in networks, which is expressed as $C_{com}$.
- Storage overhead: the average amount of copies of the time-location claims or detection information that need to be stored in a sensor when the protocol for detecting node clone attacks works in networks, which is expressed as $C_{mem}$.
- Computation overhead: the average amount of public key signing and verification operation for each node, which is denoted as $C_{cp}$.

In the ESHT scheme, the communication overhead $C_{com}$ is calculated as:

(45) $$C_{com}=C_{t}+C_{f}+C_{e}$$

where $C_{t}$ is the communication overhead of receiving time-location claim requests from the encountered track nodes, and $C_{f}$ is the communication cost of replying time-location claims to the track nodes, $C_{e}$ is the communication cost of forwarding the detection information between the trace node.

The probability that each node encounters i trace nodes in one epoch is $P_{t}(i)$:

(46) $$P_{t}(i)=\frac{\left(\begin{array}{c} \sqrt{N}\\ i \end{array}\right)\left(\begin{array}{c} N-\sqrt{N}\\ pN-i \end{array}\right)}{\left(\begin{array}{c} N\\ pN \end{array}\right)}$$

where p is the chance that any two nodes encounter in one epoch, N denotes the amount of sensor nodes in the mobile wireless sensor networks, pN denotes the average number of nodes which one sensor node encounters in one epoch . We assume a sensor node encounters i witnesses in one epoch, and then this sensor node would receive i time-location claim requests and reply i time-location claims to those witnesses. Assume E(i) is the average amount of packets which are transmitted and received by each node when a sensor encounters its witnesses:

(47) $$E(i)=\sum_{i=0}^{\sqrt{N}}2i\times P_{t}(i)$$

Substituting Equations (46) into (47) to obtain $C_{t}$:

(48) $$C_{t}=E(i)=2p\times \sqrt{N}$$

The probability that each node encounters j traced nodes in one epoch is $P_{f}(j)$:

(49) $$P_{f}(j)=\frac{\left(\begin{array}{c} \sqrt{N}\\ j \end{array}\right)\left(\begin{array}{c} N-\sqrt{N}\\ pN-j \end{array}\right)}{\left(\begin{array}{c} N\\ pN \end{array}\right)}$$

In the same way, the communication cost of replying time-location claims to the track nodes is obtained as:

(50) $$C_{f}=E(j)=2p\times \sqrt{N}$$

The probability that any two sensor nodes have k same tracked nodes in one epoch is $P_{e}(k)$:

(51) $$Pe(k)=\frac{\left(\begin{array}{c} \sqrt{N}\\ k \end{array}\right)\left(\begin{array}{c} N-k\\ \sqrt{N}-k \end{array}\right)\left(\begin{array}{c} N-\sqrt{N}\\ \sqrt{N}-k \end{array}\right)}{{\left(\begin{array}{c} N\\ \sqrt{N} \end{array}\right)}^{2}}$$

We assume two sensor nodes have k same tracked nodes. One of them would send or receive k detection information to another, so we assume E(k) is the average number of detection requests:

(52) $$E(k)=\sum_{k=0}^{\sqrt{N}}k\times P_{e}(k)$$

Substituting Equation (51) into (52) to obtain Ce:

(53) E(k)=1

Since one sensor node encounters an average of pN nodes, so the average number of sending or receiving packets including detection request and detection information is Ce:

(54) Ce=p×N×E(k)=p×N

Substituting Equations (48), (50) and (54) into (36) to obtain $C_{com}$:

(55) $$C_{com}=p\times (N+4\sqrt{N})$$

so the communication cost of ESHT scheme is O(N). In our protocol, each node needs to use a digital signature when it sends a packet. Conversely, as soon as a node retrieves a claim, it is needed to verify up to the signature. So the computation cost is in proportion to the communication cost, the computation cost is O(N).

In the ESHT scheme, in order to detect replica attacks, each witness node need to obtain sample, a sample $v_{L}^{i}$ is computed from two consecutive time-location claims of node $u_{L}$, according to Equation (3), when a sample $v_{L}^{i}$ is retrieved, the former time-location claim $C_{L}^{i-1}$ is abandoned, and only present time-location claim $C_{L}^{i}$ is stored to wait for the next time-location claim $C_{L}^{i+1}$. At the same time, to detect replica attacks in the third stage, a queue in which the detection information is stored is maintained. So the fixed length of storage space including a time-location and a queue is required for each node. Every node has $\sqrt{N}$ tracked nodes. Conversely, every node has $\sqrt{N}$ trace nodes, so the storage cost of every node is $O(\sqrt{N})$. The comparison of system overhead between [15,24,25] and our proposed scheme is summarized in Table 3.

An analogue test is carried out to test the feasibility and accuracy of the scheme by using OMNeT++ platform. OMNeT++ is a scalable, modular simulink and framework, mainly for constructing network emulators. In the testing, N sensor nodes distribution are relatively uniform within a square area of size 1000 m × 1000 m, where N varies from 100 to 1000. The communication range of each node varies from 50 m to 100 m. The Random Waypoint Mobility Model (RWP) is adopted as the movement model. We use the code of Ganeriwal et al. [41] to construct the movement model based on Random Waypoint Mobility mode with steady-state distribution.

In the movement model, every node randomly selects a speed which is in the interval of 2~8 m/s to move toward a specific waypoint. At the close of each speech, a node stops for a random duration $T_{stop}$ after moving for T unit time, where $T_{stop}$ varies from 0 s to 20 s.

In the simulation experiment, only one comprised node and its one replica are deployed in the network. The communication between different nodes applies the standard unit-disc two-way communication pattern. At the same time, the IEEE 802.11 protocol is adopted as the medium access control protocol for each node. Assume the user-configured false negative rate γ=0.01 and the user-configured false positive rate η=0.01. Every experiment is carried out for 1000 simulation seconds, and the average value of 10 experimental results is discussed.

**Figure 1:**
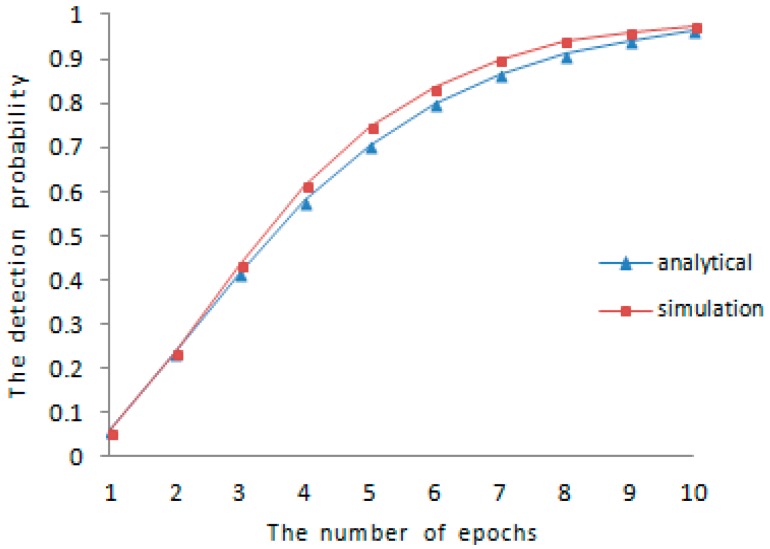
Comparison of detection probability (R=40, N=1000).

**Figure 2:**
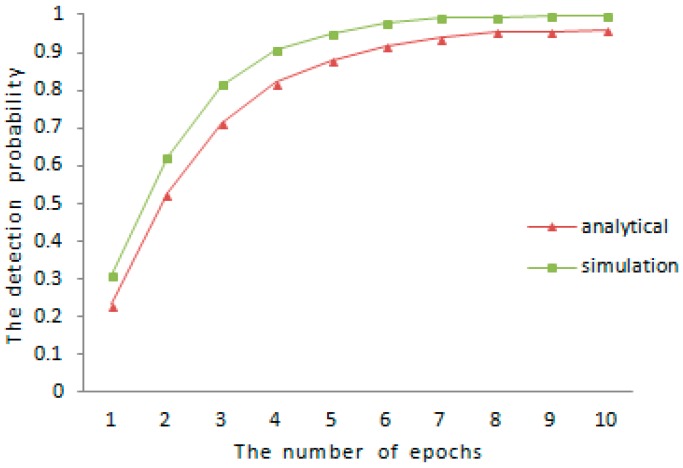
Comparison of detection probability (R=100, N=500).

The lower bound of detection probability is analyzed in the earlier part of Section 5, the comparison between theoretical analysis and experimental results for the detection probability is shown in Figure 1 and Figure 2, respectively. We vary the amount of mobile sensor nodes in sensor networks and the communication range between different sensor nodes. When N=1000 and R=40, the result of comparison is shown in Figure 1. When N=500 and R=100, the result of the comparison is shown in Figure 2. As is shown in both of Figure 1 and Figure 2, the experimental detection probability is higher than the low bound of detection probability discussed before.

**Figure 3:**
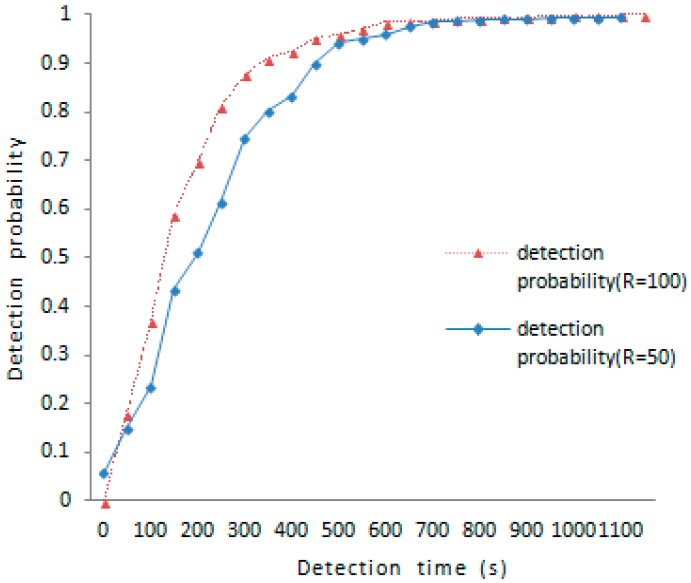
Detection probability versus detection time.

The detection time is an important metric to evaluate the proposed scheme. Because the communication range reflects the encounter probability, and the encounter probability decides the time elapsed between each meeting, so that we would discuss the change of detection probability with over time on the effects of different communication ranges. As is shown in Figure 3, the detection probability under R=50 is 51.1% and the detection probability under R=100 is 82.1% about 250 s later; the detection probability under R=100 is above 90% about 350 s later. To achieve the same result in the case of R=50, it takes at least 550 s to reach 90% detection probability. So the larger the communication range, the higher the detection probability.

**Figure 4:**
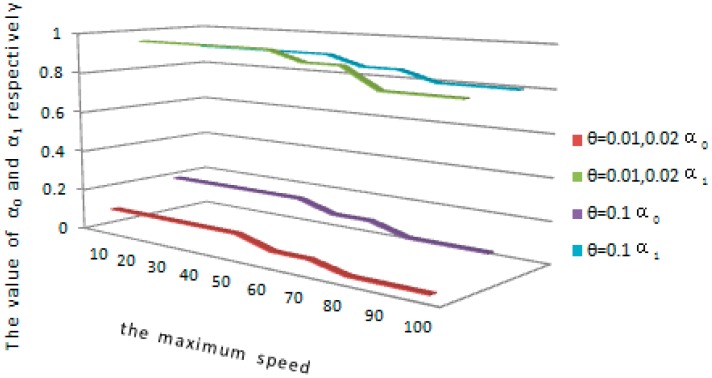
The configurations of $\alpha_{0}$ and $\alpha_{1}$.

Simulations are conducted to demonstrate the impact of time synchronization and localization errors on the proposed protocol. We use ideal temporal synchronization and positioning method to measure the speed, and then the measured speed *v* is modified as *v*′, *v*′ is selected uniformly at random from the range of v−vθ and v+vθ, where θ is defined as the maximum speed error rate. $\alpha_{0}$ and $\alpha_{1}$ are set in accordance with the maximum speed error rate. Figure 4 shows how to take different values of $\alpha_{0}$ and $\alpha_{1}$ according to the maximum speed $v_{\max}$ which is scientifically configured by the system. As shown in Figure 4, when the system-configured maximum speed $v_{\max}$ is in the interval of 10~60 m/s, and the maximum speed error rate is 0.01 or 0.02. $\alpha_{0}$ and $\alpha_{1}$ are set to 0.1 and 0.95 respectively. When the predefined maximum value $v_{\max}$ is in the interval of 60~80 m/s and the maximum speed error rate is 0.01 or 0.02, $\alpha_{0}$ and $\alpha_{1}$ are set to 0.05 and 0.9 respectively. When the system-configured maximum speed $v_{\max}$ is in the interval of 80~100 m/s and the maximum speed error rate is 0.01 or 0.02, $\alpha_{0}$ and $\alpha_{1}$ are set to 0.01 and 0.8 respectively. When the system-configured maximum speed $v_{\max}$ is in the interval of 10~60 m/s and the maximum speed error rate is 0.1, $\alpha_{0}$ and $\alpha_{1}$ are set to 0.2 and 0.9 respectively. When the predefined maximum value $v_{\max}$ is in the range of 60 to 80 and the maximum speed error rate is 0.1, $\alpha_{0}$ and $\alpha_{1}$ are set to 0.15 and 0.85 respectively. When the system-configured maximum speed $v_{\max}$ is in the interval of 80~100 m/s and the maximum speed error rate is 0.1, $\alpha_{0}$ and $\alpha_{1}$ are set to 0.1 and 0.8 respectively. So it is deduced that the configurations of $\alpha_{0}$ and $\alpha_{1}$ are inversely proportional to variety in the system-configured maximum speed $v_{\max}$.

**Figure 5:**
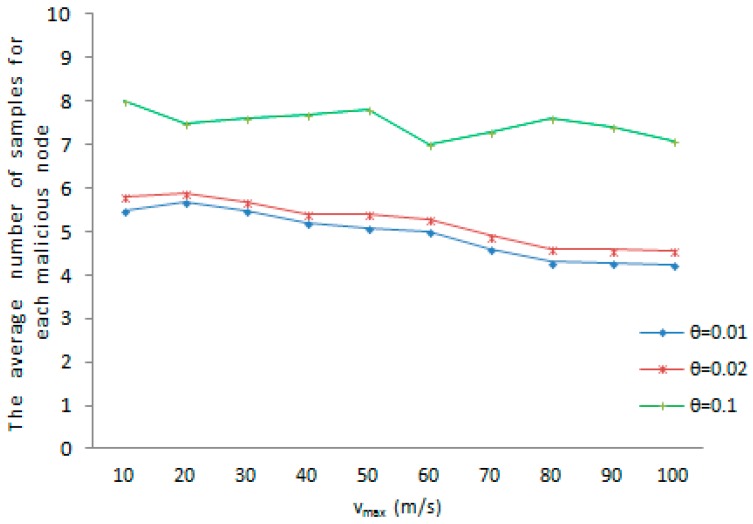
The average number of samples for each malicious node.

**Figure 6:**
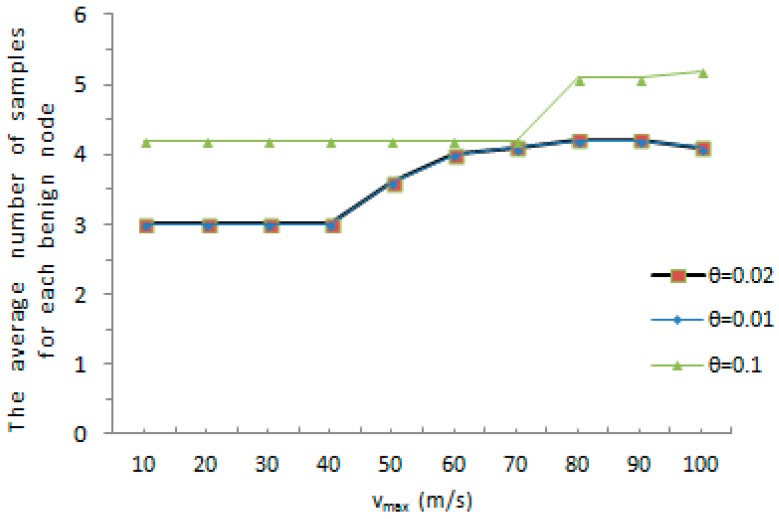
The average number of samples for each benign node.

The average amount of samples for every tracked node is the amount of samples required for the witness node to judge whether a node has been cloned or not. We evaluate the average number of samples for each tracked node under different system-configured maximum speed when a malicious node is accurately found out as cloned node. As shown in Figure 5, the average amount of samples achieves a maximum value at 8, when the maximum speed $v_{\max}=10$ and the maximum speed error rate θ=0.1. The average number of samples reaches a minimum value at 4.25 when the predefined maximum value $v_{\max}=100$ and the maximum speed error rate θ=0.01. There is another dimension, Figure 6 shows the average amount of samples for each tracked node under different maximum speed when a benign node is accurately detected as a normal node. As shown in Figure 6, the average amount of samples achieves a maximum value at 5.2, when the predefined maximum speed $v_{\max}=100$ and the maximum speed error rate θ=0.1. The average amount of samples reaches a minimum value at 3 when the predefined maximum speed $v_{\max}=10$ and the maximum speed error rate θ=0.01. As the whole, with the increase of the predefined maximum value, the average number of samples for each tracked node rises or drops slightly. The witness node would detect whether a mobile sensor node has been replicated or not with a smaller number of samples in both cases. Meanwhile, it is obvious that an increase of the maximum speed error rate θ results in the growth of the average amount of samples for each tracked node. It can be further reasoned that the faster the movement speed, the higher the chance that the measured speed of a normal node is erroneous detected to be over the predefined maximum speed. On the contrary, the faster the movement speed, the less chance that the measured speed of a malicious node is erroneous detected to be below the system-configured maximum speed.

**Figure 7:**
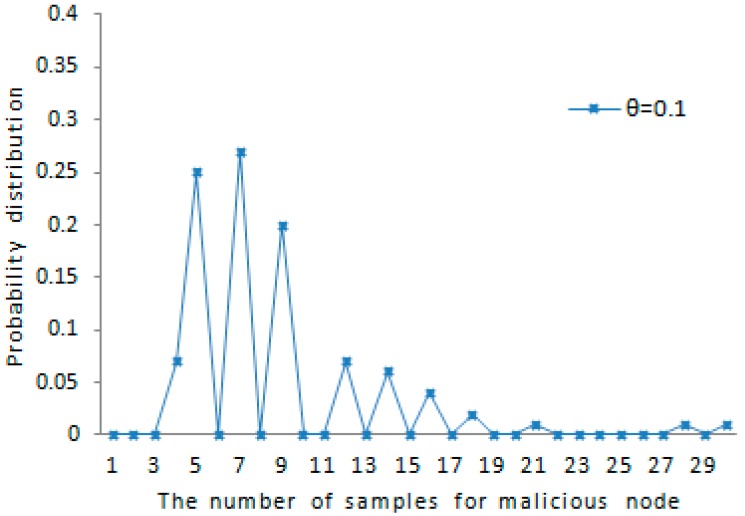
The probability distribution of the amount of samples for malicious node.

**Figure 8:**
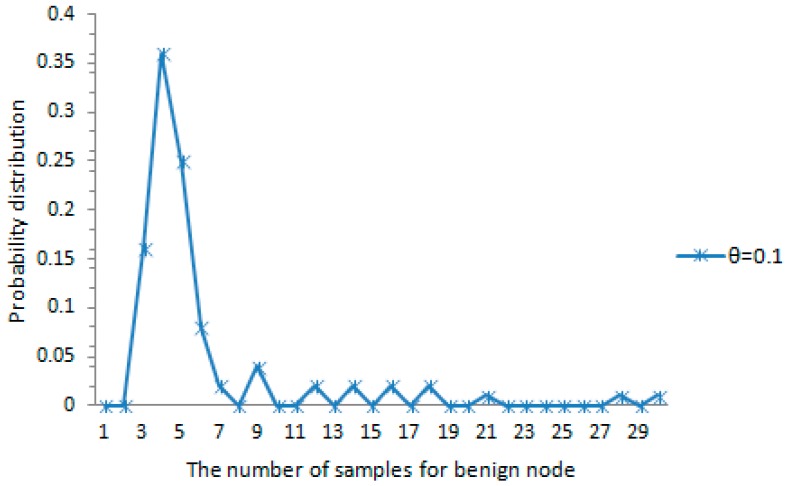
The probability distribution of the amount of samples for benign node.

The probability distribution of the amount of samples for each malicious node detected accurately is shown in Figure 7. When the maximum speed error rate θ=0.1 and the maximum speed $v_{\max}=20$, about 79% of all the case falls in the range of [4,9] as shown in the figure. This also indicates that the probability distribution of the amount of samples satisfies the rule reflected in Figure 5. The amount of samples for each tracked node is less than or close to the average amount for each tracked node. The probability distribution of the amount of samples for each benign node detected accurately is shown in Figure 8. When the maximum speed error rate θ=0.1 and the system-configured maximum speed $v_{\max}=20$, about 87% of all the case falls in the range of [3,7] as shown in the figure. This also indicates that the probability distribution of the amount of samples satisfies the rule reflected in Figure 6. The amount of samples for each benign node is below or close to the average amount for each benign node. As we can see from Figure 5 and Figure 6, it is obvious that whether a mobile sensor node is malicious node can be decided quickly with a few samples for each tracked node.

**Figure 9:**
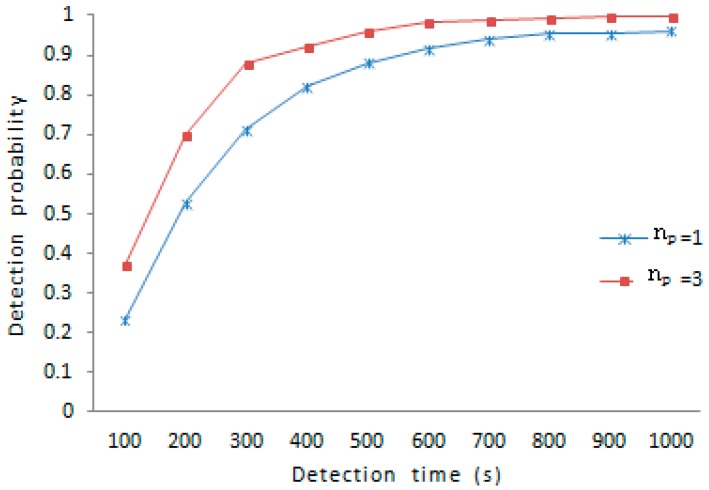
The detection probability under different $n_{p}$.

In the process of detection, one queue for each tracked node in the tracking set is maintained, which can hold more than two corresponding detection information. Because the more samples are measured, the more accurate the detection, the length of the queue denoted as $n_{p}$ is set to a higher value to accelerate the speed of detection. But the longer the queue, the more space costs, to avoid overload for the tracking node, therefore making the size of each queue equal to 3 is meeting demand. As is shown in Figure 9, under the same amount of nodes compromised, the scheme with $n_{p}=3$ shows stronger resilience than the scheme with $n_{p}=1$. For example, the detection probability under $n_{p}=3$ is 37.1% and the detection probability under $n_{p}=1$ is 23.3%, when the detection time is 100 s; the detection probability under $n_{p}=3$ is 95.9% and the detection probability under $n_{p}=1$ is 88.1%, when the detection time is 500 s. At the beginning of detection, the growth rate of detection probability under $n_{p}=3$ is higher than that of detection probability under $n_{p}=1$. About 400 s later, the growth rate of detection probability under $n_{p}=3$ is gradually slower than that of detection probability under $n_{p}=1$. Generally speaking, the detection probability under $n_{p}=3$ is higher than the detection probability under $n_{p}=1$ just as shown in Figure 9.

**Figure 10:**
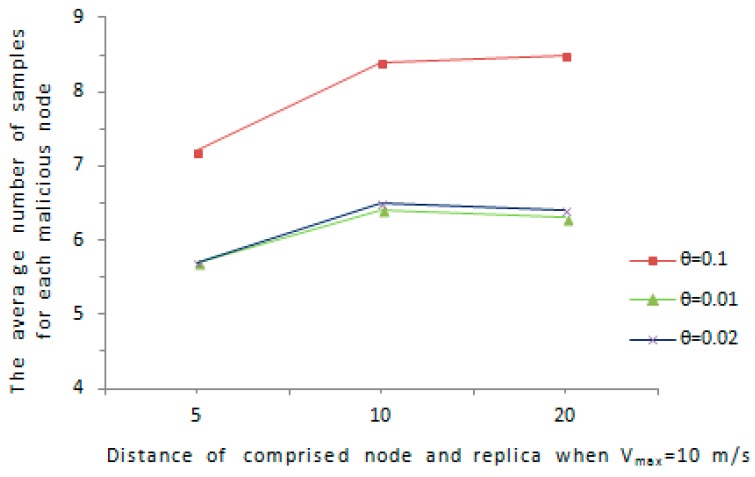
The average number of samples versus D when *V*_max_ = 10 m/s.

**Figure 11:**
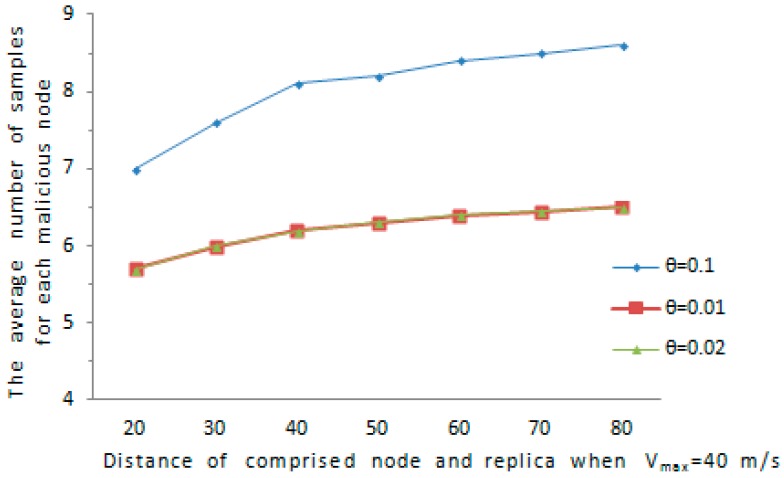
The average number of samples versus *D* when *V*_max_ = 40 m/s.

We further consider a special case, if the comprised node and its replica remain static at a certain distance, it is possible for the malicious node to avoid being discovered, for example, the comprised node and its replica are not detected with high probability when D≤Dmax2 and $V_{\max}=D_{\max}/s$, because the detection is based on speed. Assume D indicates the distance between the comprised node and the cloned node, we evaluate the distance D in such a way that D varies from Dmax2 to $2D_{\max}$, where $D_{\max}$ is set according $V_{\max}$. We evaluate the detection ability of our proposed protocol in the case of D is relatively short. We consider the D is in the interval of [Dmax2,2Dmax] and $V_{\max}$ is in the range of [10,50]. As is shown in Figure 10 and Figure 11, the average amount of samples for malicious node increases with the maximum speed error rate θ. As far as the affect of D on the amount of samples for a abnormal node, when D grows from Dmax2 to $2D_{\max}$, the average amount of samples for an abnormal node increases obviously. Overall, the average amount of samples for an abnormal node is below 9.5 in all cases, and the comprised node and its replica can be detected with a reasonable amount of samples.

**Figure 12:**
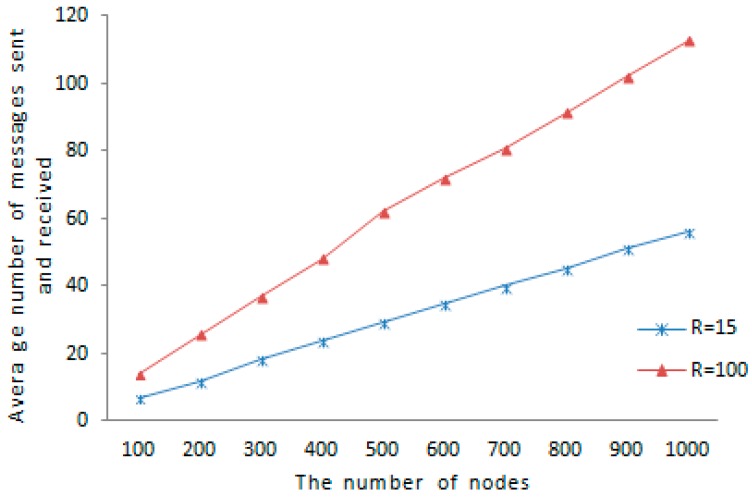
Communication overhead.

In our scheme, the average amount of messages that each node transmitted and received in one epoch is utilized to estimate the communication overhead. Figure 10 shows that the communication overhead changes over the number of nodes uniformly distributed in wireless sensor networks. The communication overheads under different communication ranges are compared. When the communication range of sensor node increases, the set of node’ one-hop neighbors enlarges. The probability that two tracking nodes with same tracked node encounter increases, and the detection information forwarded to witness node for detection increases accordingly, so the communication overhead becomes higher. As is shown in Figure 12, the average amount of messages sent and received under R=100 is 62, and the average amount of messages sent and received under R=15 is 29, as the network size is 500 nodes. The average amount of messages sent and received under R=100 is 113 and the average amount of messages transmitted and received under R=15 is 56 when the network size is 1000 nodes, so the larger the communication range, the higher the communication overhead, further a gap of the average amount of messages transmitted and received under the two communication range becomes more and more large with the network pattern’s augmentation.

## 6. Conclusions

In the light of the mobility feature of nodes in mobile network environments, a novel distributed detection protocol for detecting mobile node replication attacks in mobile networks has been proposed. In the Encounter-based Sequential Hypothesis Testing scheme (ESHT), the encounter between different nodes is made full use of, and sensor’s time-location claims are forwarded to obtain samples for detection when the corresponding tracking nodes meet. Meanwhile, Sequential Tests of Statistical Hypotheses are applied to further detect the cloned nodes using witness nodes. This is able to resist the smart attacks of cloned node. On the one hand, the overhead of maintaining traditional multi-hop routing path is saved to achieve energy saving effects, while on the other hand, our simulation shows that whether a mobile sensor node is malicious node can be decided quickly with a small amount of samples for each tracked node. At the same time, the false positive rate and false negative rates are low. Theoretical analysis and empirical results demonstrate the success probability of clone detection is high enough. Regarding communication cost and memory cost, the system overhead of our scheme is reasonable. In the future work, the performance of our scheme could be evaluated when applied in other mobility models. Beyond that, various kinds of attacks such as witness-void replication attacks which are directly against our scheme and the related policy which can defend these attacks would be studied.

## Figures and Tables

**Table 1:** Symbols and notations.

Symbol	Denotation
*R*	Transmission radius of node.
*X_i_*(*t*)	Position of node *i* at time *t*.
Epoch	The procedure during which a node moves to somewhere at the same rate, and in the same direction, and then stops for a while.
*L*	The length of an epoch, which is the distance from the point of epoch beginning to the point of epoch ending.
*ν*	The speed of node in an epoch, which is distributed in the interval $[v_{\min},v_{\max}]$, $\bar{v}$ is the mean speed.
*T_stop_*	The random residence time selected by node when an epoch is over, the length of residence time is randomly distributed in the interval [0,*T*_max_], ${\bar{T}}_{stop}$ is the mean value of $T_{stop}$
$\bar{T}$	The average length of time that node spends in movement status before an epoch is over. T¯=L¯V¯, the total time is $\bar{T}+{\bar{T}}_{stop}$.
*ν_mm_*	The relative speed between node *i* and node *j* when they move in movement mode mm, $v_{mm}=\left|{\bar{v}}_{i}-{\bar{v}}_{j}\right|$, where $\bar{vi}$ is the velocity vector of the node *i*.
${\hat{v}}_{mm}$	The standard relative speed of movement mode mm. v^mm=v¯mmv¯.

**Table 2:** Notations used in the proposed protocol.

Notation	Denotation
$v_{\max}$	The predefined maximum speed.
$\alpha_{0}$	The possibility of the event that a normal node is misjudged as replica due to temporal synchronization and positioning errors.
$\alpha_{1}$	The possibility of the event that the measured speed of a replica exceeds the predefined maximum value $v_{\max}$.
η	The maximum false positive rate.
γ	The maximum false negative rate.
$I_{a}^{L}$	The detection information denoted as $\left(n_{a}^{L},\omega_{a}^{L}\right)$.
$v_{L}^{i}$	The measured speed at a time $T_{i+1}$ to be trial sample in Bernoulli trial.
$n_{p}$	The length of the queue for each tracked node.
$n^{x}$	The total number of speed samples of the tracked node x.
$\omega^{x}$	The cumulative total of the amount of times a specific event that $S_{x}^{i}=1$ occurs in the $n^{x}$ samples of the tracked node x.

**Table 3:** Comparison with [15,24,25].

	Yu et al. [15]	Ho et al. [24]	Manickavasagam et al. [25]	Proposed Scheme
Method	Distributed	Centralized	Distributed	Distributed
Communication overhead	$O(N^{2})$	$O(N\sqrt{N})$	O(N)	O(N)
Computation overhead	/	O(N)	$O(\sqrt{N})$	O(N)
Storage overhead	O(N)	O(1)	O(N)	$O(\sqrt{N})$
